# Potential Adverse Effect of Nonsteroidal Anti‐Inflammatory Drugs (NSAIDs) on Bisphosphonate Efficacy: An Exploratory Post Hoc Analysis From a Randomized Controlled Trial of Clodronate

**DOI:** 10.1002/jbmr.4548

**Published:** 2022-04-20

**Authors:** Zhangan Zheng, Helena Johansson, Nicholas C. Harvey, Mattias Lorentzon, Liesbeth Vandenput, Enwu Liu, John A. Kanis, Eugene V. McCloskey

**Affiliations:** ^1^ Mellanby Centre for Musculoskeletal Research, Medical Research Council (MRC) Versus Arthritis Centre for Integrated Research in Musculoskeletal Ageing, Department of Oncology & Metabolism University of Sheffield Sheffield UK; ^2^ Department of Trauma and Spine Surgery The Second People's Hospital of Wuhu Wuhu China; ^3^ Sahlgrenska Osteoporosis Centre, Department of Internal Medicine and Clinical Nutrition, Institute of Medicine Sahlgrenska Academy, University of Gothenburg Gothenburg Sweden; ^4^ Mary McKillop Institute for Health Research Australian Catholic University Melbourne VIC Australia; ^5^ MRC Lifecourse Epidemiology Centre University of Southampton Southampton UK; ^6^ Centre for Metabolic Bone Diseases University of Sheffield Sheffield UK

**Keywords:** NSAID, fracture, BMD, bisphosphonate, clodronate

## Abstract

Nonsteroidal anti‐inflammatory drugs (NSAIDs) have been reported to have weak but beneficial effects on bone health, including fracture risk, but many epidemiological studies are likely confounded. We explored the relationship between NSAIDs and fracture risk in a post hoc analysis of a well‐documented, randomized, placebo‐controlled study of the bisphosphonate, clodronate, in which treatment reduced osteoporotic fracture risk by 23%. Concurrent medication use at baseline was used to identify those prescribed oral NSAIDs. Only verified, incident fractures were included in the analysis. A total of 1082 (20.8%) women reported use of NSAIDs at baseline. They were slightly, but significantly, younger (mean 79 versus 80 years, *p* = 0.004), heavier (mean 66.7 versus 64.7 kg, *p* < 0.001) than nonusers, with slightly higher femoral neck bone mineral density (FN‐BMD, 0.66 versus 0.64 g/cm^2^, *p* < 0.001). In an adjusted model, NSAID use was associated with a significant increase in osteoporotic fracture risk over the 3‐year study period (hazard ratio [HR] 1.27; 95% confidence interval [CI], 1.01–1.62; *p* = 0.039). However, this increase in risk was not statistically significant in the placebo group (HR 1.11; 95% CI, 0.81–1.52). In women receiving clodronate, the effect of the bisphosphonate to reduce osteoporotic fracture risk was not observed in those receiving NSAIDs (HR 0.95; 95% CI, 0.65–1.41; *p* = 0.81) in contrast to those not using NSAIDs (HR 0.71; 95% CI, 0.58–0.89; *p* = 0.002). In a subset with hip BMD repeated at 3 years, BMD loss during clodronate therapy was greater in those women receiving NSAIDs than in nonusers (eg, total hip −2.75% versus −1.27%, *p* = 0.078; femoral neck −3.06% versus −1.12%, *p* = 0.028), and was not significantly different from that observed in women receiving placebo. The efficacy of the bisphosphonate, clodronate, to reduce fracture risk was largely negated in those receiving NSAIDs. Although the mechanism is unclear, this clinically significant observation requires exploration in studies of commonly used bisphosphonates. © 2022 The Authors. *Journal of Bone and Mineral Research* published by Wiley Periodicals LLC on behalf of American Society for Bone and Mineral Research (ASBMR).

## Introduction

Nonsteroidal anti‐inflammatory drugs (NSAIDs) are one of the most commonly prescribed medications for various acute and chronic musculoskeletal or inflammatory diseases. In European countries, NSAIDs account for ~5% to 11% of all medications prescribed every year.^(^ ^1^ ^)^ In the United States, a recent survey showed that 63% of adults reported having used NSAIDs within the past 12 months.^(^ ^2^ ^)^ Among elderly patients >65 years, the prevalence of ever NSAID use could reach at as high as 96.4%, and the proportion exposed to chronic use (ie, took NSAIDs regularly for more than 1 month) was 32.9%.^(^ ^3^ ^)^

NSAIDs commonly exert their anti‐inflammatory and analgesic properties by inhibiting the synthesis of prostaglandins via cyclooxygenase enzymes (COX).^(^ ^4^, ^5^ ^)^ Because prostaglandins, particularly prostaglandin E2 (PGE2) and prostaglandin I2 (PGI2), are involved in bone remodeling,^(^ ^6^, ^7^, ^8^ ^)^ the relationship between NSAIDs use and fracture risks has been widely reported in the literature.^(^ ^9^, ^10^, ^11^, ^12^, ^13^, ^14^ ^)^ However, conclusions derived from these studies are conflicting and variously suggest that NSAIDs use is a protective factor,^(^ ^9^, ^10^ ^)^ a risk factor for fracture,^(^ ^11^, ^12^, ^13^ ^)^ or has no effect on fracture risk.^(^ ^14^ ^)^ Thus, the true impact of NSAIDs use on fracture risks, if any, remains unclear, reflecting the inability to adjust for other fracture‐related risk factors in most of these studies.

Bisphosphonates remain the most widely used therapeutic class in the treatment of various metabolic bone diseases associated with excessive bone resorption, particularly osteoporosis. Clodronate, a first‐generation, non‐nitrogen‐containing bisphosphonate, has shown efficacy in reducing fracture risk in osteoporosis, skeletal events in metastatic breast cancer and myelomatosis, and the prevention of bone metastases in early breast cancer.^(^ ^15^, ^16^, ^17^, ^18^, ^19^, ^20^, ^21^, ^22^, ^23^ ^)^ Following a pilot study, we conducted a single‐center, prospective, randomized, placebo‐controlled study of clodronate 800 mg in community‐dwelling women age ≥75 years, with the purpose of identifying risk factors for fracture and determining the efficacy of an intervention to reduce fracture risk in women unselected for bone mineral density (BMD)‐defined osteoporosis. Treatment with clodronate was associated with a 23% reduction in osteoporotic fracture risk over 3 years.^(^ ^19^ ^)^ The comprehensive collection of data on fracture risk and other information captured systematically as part of the trial, including concomitant medications, enabled an exploration of the hypothesis that NSAID use influences future fracture risk.

## Subjects and Methods

### Study subjects

The study design and characteristics of the women recruited to the study have been published.^(^ ^19^, ^24^, ^25^, ^26^ ^)^ In brief, we recruited elderly community‐dwelling women (≥75 years) from Sheffield and the surrounding region; potential participants were identified using local general practice lists and were sent a letter of invitation. In total, over 35,000 elderly women were approached, and 5212 were recruited to the main study (380 women recruited to a pilot feasibility study are not included in this analysis). Inclusion criteria were broad (female age ≥75 years and willing to participate), with a relatively small number of exclusion criteria. The latter comprised concurrent treatment for a malignancy; concurrent medication likely to influence skeletal metabolism (except calcium supplements 500 mg daily); bilateral hip arthroplasties; known malabsorptive states; and impaired mental state or concurrent illness that would impede informed consent or compliance with the study. At the screening visit, women were also excluded if there was evidence of significant impairment of renal or hepatic function (serum creatinine >300μM or alanine aminotransferase [ALT] ≥ twice the upper limit of the reference range, respectively), or underlying metabolic bone disease (eg, osteomalacia); evidence of calcium disorders also meant exclusion apart from mild primary hyperparathyroidism (adjusted serum calcium <3.0 mmol/L). All participants gave fully informed written consent for use of the collected data; the study was approved by the local Ethics Committee.

### Baseline information including medication use

Detailed and comprehensive assessments of each participant were carried out during the screening visit in the then World Health Organization (WHO) Collaborating Centre for Metabolic Bone Diseases in Sheffield, UK, including date of birth, blood pressure, pulse rate, medical history, height (m), weight (kg), current medication, and supplement use. Weight was measured on a digital scale (TanitaBWB‐600, Tanita Corporation, Tokyo, Japan), wearing indoor clothing without shoes. Height was measured using Harpenden stadiometer (Holtain Ltd, Crymych, Dyfed, UK). Body mass index (BMI) was calculated using weight/height^2^ (kg/m^2^). Furthermore, participants reported their medical history with specific questions capturing self‐reports of previous stroke, diabetes, osteoarthritis, hypertension, thyroid disease, Parkinson's disease, and rheumatoid arthritis.

Participants were specifically questioned at the screening visit by study nurses about the medication (including over‐the‐counter [OTC] drugs and supplements) used in the month prior to study entry, checked against a general practitioner (GP) prescription where available, and included an estimate of the year that any such treatment had started to estimate duration of use. The use of NSAIDs was defined as using any kind of NSAID at baseline, excluding low‐dose aspirin (<75 mg/d) and paracetamol. Concurrent exposure to other commonly used medications was classified using the following categories: angina/anti‐arrhythmia, antihypertensives (beta blockers, angiotensin converting enzyme [ACE] inhibitors, calcium‐channel blockers, diuretics), statins, antacids (histamine type 2 receptor antagonists [H2RAs], proton pump inhibitors [PPIs], other), laxatives, asthma and/or chronic obstructive pulmonary disease (COPD), diabetes mellitus, anti‐epileptics, psycholeptics (antidepressants, anxiolytics), oral glucocorticoids, and thyroid drugs.

### Baseline tests

At baseline, venous blood samples were collected from each participant, between 8:00 a.m. and 10:00 a.m. after an overnight fast, to measure the serum levels of routine blood hematological and biochemical parameters, including renal function, liver function including total alkaline phosphatase (ALP), calcium, and erythrocyte sedimentation rate (ESR). Subsequently, the samples were analyzed for the bone formation marker, serum procollagen N‐terminal peptide of type 1 collagen (P1NP), and the bone resorption marker, serum C‐telopeptide of type 1 collagen (CTX), using the Elecsys® total P1NP and β‐CrossLaps, respectively, on a Cobas e411 automated immunoassay (Roche Diagnostics, Penzberg, Germany). The interassay coefficients of variation (CVs) were 3.8% and 5.5%, respectively.

BMD was measured by dual‐energy X‐ray absorptiometry (DXA) at the total hip and subregions using a Hologic QDR4500 Acclaim densitometer (Hologic, Inc., Marlborough, MA, USA). The same device was used to image the lateral thoracic and lumbar spines to detect vertebral fractures using vertebral morphometry. Finally, distal forearm BMD was also measured using DXA (Osteometer DTX 100; Osteometer AS, Copenhagen, Denmark).

### Study treatment, follow‐up, and outcomes

After randomization, the women received either clodronate 800 mg daily (two BONEFOS 400‐mg tablets once daily or one tablet twice daily) or an identical placebo. Study medication was taken on an empty stomach with a drink of water at least 1 hour before breakfast. It could also be taken in the middle of the night if desired by the women after fasting for ∼5–6 hours. Concomitant calcium and vitamin D supplementation was not given. The intervention was continued for 3 years, the timeframe for this analysis. The dates of treatment start and finish, as opposed to study start and finish, were recorded and 6‐monthly compliance with treatment was undertaken by pill counts with unused medication being collected at the end of each 6‐month period. These data therefore required the participant to complete the first 6 months of the study.

Incident fractures and hospital admissions were inquired about at each visit from the point of randomization until the end of follow‐up. All reported fractures were confirmed by hospital notes, discharge/general practitioner letters, copies of radiographic reports, or review of radiographs if necessary. Only verified fractures at any anatomical site within the follow‐up period were included as outcomes. Incident osteoporotic fractures were defined as those excluding fractures of the skull, nose, face, hand, finger, feet, toe, ankle, and patella fractures.^(^ ^27^ ^)^

Repeat BMD measurements at the hip were undertaken at the end of the 3‐year intervention period in a small subset of the women. This comprised a largely random sample complemented by an enriched subsample from those with either primary hyperparathyroidism or vertebral fractures (detected on DXA lateral spine images) at entry to the study, reflecting research interests in the department at the time. The repeat measurements were conducted on the same Hologic scanner as used at baseline.

### Statistical analysis

This was a post hoc analysis. Baseline characteristics in NSAID users and nonusers were expressed as mean ± standard deviation (SD) or proportion, with appropriate use of one‐way analysis of variance (ANOVA) or chi‐square tests. Cox regression analyses were used to investigate the effect of NSAIDs use on fracture incidence in univariate and adjusted models, with adjustments including variables that were significantly different between NSAID users and nonusers, and treatment group (clodronate/placebo) when analyses were conducted in the whole study population. An interaction term NSAID × treatment group was also examined in unadjusted and adjusted models. Univariate and adjusted models were also examined separately in the placebo and clodronate groups. Cox regression was also used to examine the efficacy of clodronate versus placebo in NSAID users and nonusers. Percentage changes in BMD at the hip and subregions were examined using ANOVA.

The distribution of compliance (medication taken/medication provided) with clodronate was non‐Gaussian and statistical analysis comprised a comparison of median intakes using the Mann‐Whitney *U* test. In addition, we used an approach whereby those taking more than 80% of expected medication over the treatment duration were categorized as high compliance, and those taking 80% or less as low compliance. These categories were compared using chi‐square tests.

Values of *p* ≤ 0.05 were considered statistically significant. All statistical analyses were conducted using IBM SPSS Statistics, version 25 (IBM Corp., Armonk, NY, USA).

## Results

### Baseline characteristics

Of the 5212 women recruited to the study, 1082 (21%) reported current use of NSAIDs at study entry. Among NSAIDs users, the most commonly reported drugs were ibuprofen (*n* = 357) and diclofenac (*n* = 311) (Table S1). The baseline characteristics of NSAIDs users and nonusers are shown in Table 1. In users, the median duration of exposure to NSAIDs was 4 years (interquartile range [IQR], 1–8 years). Compared with nonusers, NSAIDs users were slightly but statistically significantly younger and heavier, with more frequently self‐reported histories of osteoarthritis or rheumatoid arthritis (Table 1, Table S1). Additionally, NSAIDs users were more likely to self‐report intake of vitamin D supplements (50.6% versus 45.5%, *p* = 0.002), with prescription data showing significantly lower use of asthma/COPD medications (10.0% versus 13.2%, *p* = 0.004), but increased exposure to psycholeptic medications (26.9% versus 22.7%, *p* = 0.004) (Table 1, Table S1). The use of antacid medications was similar in NSAID users and nonusers (26.0% versus 25.6%, respectively), though there were small but significant differences in the types of antacid medication. For example, at baseline NSAID users had a slightly higher use of histamine H2‐receptor antagonists (H2RA; 10.4% versus 8.2%, *p* = 0.021) and a slightly lower use of proton pump inhibitors (PPIs; 6.7% versus 8.8%, *p* = 0.031) than nonusers. BMD values at the total hip, subregions, and distal forearm were slightly but statistically significantly higher in the NSAID users than in nonusers, whereas measures of muscle strength and performance were lower. For example, the proportion of women observed to have difficulty in the sit‐to‐stand test was higher in NSAID users than in nonusers (38.3% versus 29.5%, *p* = 0.001). Finally, there was a number of small but significant differences in mean values of routine hematological and biochemical assessments in keeping with known effects of NSAIDs on renal and hepatic function and blood counts (Table 1, Table S2).

**Table 1:** Baseline Characteristics in Women With and Without NSAIDs Use, Including Proportion Randomized to Clodronate During the Study

Variable	No NSAID (*n* = 4130)	NSAID (*n* = 1082)	*p*
General characteristics, mean ± SD			
Age (years)	79.58 ± 4.02	79.19 ± 3.62	0.004
Height (m)	1.56 ± 0.06	1.56 ± 0.06	0.55
Weight (kg)	64.66 ± 12.06	66.71 ± 12.12	0.000
BMI (kg/m^2^)	26.58 ± 4.62	27.50 ± 4.64	0.000
Medical history, *n* (%)			
Osteoarthritis	2828 (68.5)	902 (83.4)	0.000
Rheumatoid arthritis	96 (2.3)	53 (4.9)	0.000
Hyperthyroidism	35 (0.8)	17 (1.6)	0.033
Prior fracture	1854 (45.4)	503 (46.8)	0.40
Falls in previous month	139 (3.6)	35 (3.5)	0.92
Medication use/condition, *n* (%)			
Calcium^a^	607 (14.7)	185 (17.1)	0.05
Vitamin D^a^	1879 (45.5)	548 (50.6)	0.002
Angina/anti‐arrhythmia	798 (19.3)	225 (20.8)	0.28
Anti‐hypertensive	2318 (56.1)	617 (57.0)	0.61
Statins	85 (2.1)	23 (2.1)	0.91
Asthma/COPD	546 (13.2)	108 (10.0)	0.004
Antacids	1058 (25.6)	281 (26.0)	0.82
PPI	363 (8.8)	73 (6.7)	0.031
Diabetes mellitus	160 (3.9)	38 (3.5)	0.66
Epilepsy	99 (2.4)	28 (2.6)	0.74
Glucocorticoids	136 (3.3)	40 (3.7)	0.51
Laxatives	539 (13.1)	146 (13.5)	0.72
Psycholeptics	936 (22.7)	291 (26.9)	0.004
Thyroid disease	356 (8.6)	102 (9.4)	0.40
Baseline biochemistry diagnoses, *n* (%)			
eGFR <30 mL/min/1.73 m^2^	141 (3.5)	59 (5.5)	0.016
Primary hyperparathyroidism	111 (2.7)	15 (1.4)	0.013
Bone mineral density (g/cm^2^), mean ± SD			
Total hip	0.75 ± 0.14	0.77 ± 0.14	0.000
Femoral neck	0.64 ± 0.12	0.66 ± 0.12	0.000
Trochanteric	0.57 ± 0.12	0.58 ± 0.12	0.006
Intertrochanteric	0.88 ± 0.17	0.90 ± 0.17	0.001
Distal forearm	0.34 ± 0.08	0.35 ± 0.08	0.002
Serum bone turnover markers (mean ± SD)			
Total ALP (IU/L)	206.34 ± 77.81	209.31 ± 70.12	0.08^b^
P1NP (ng/mL)	62.5 ± 33.4	66.8 ± 39.0	0.002^b^
CTX (ng/mL)	0.40 ± 0.21	0.41 ± 0.22	0.61^b^
Muscle strength (right limbs), mean ± SD			
Maximum quadriceps (N)	127.4 ± 60.2	121.1 ± 55.3	0.003
Maximum grip (kg)	12.4 ± 6.4	12.0 ± 6.3	0.044
Difficulty in sit‐to‐stand (%)	1217 (29.5)	414 (38.3)	0.001
Study treatment, *n* (%)			
Clodronate	2051 (49.7)	555 (51.3)	0.34

Additional information is provided in Tables S1 and S2.

ALP = alkaline phosphatase; CTX = C‐terminal telopeptide of collagen type I; eGFR = estimated glomerular filtration rate; OTC = over the counter; P1NP = procollagen type I N‐propeptide.

^a^ Data includes self‐reported use of OTC medications.

^b^ Statistical comparison based on log‐transformed data.

### Relationship between NSAID use and osteoporotic fracture risk

**Fig. 1:**
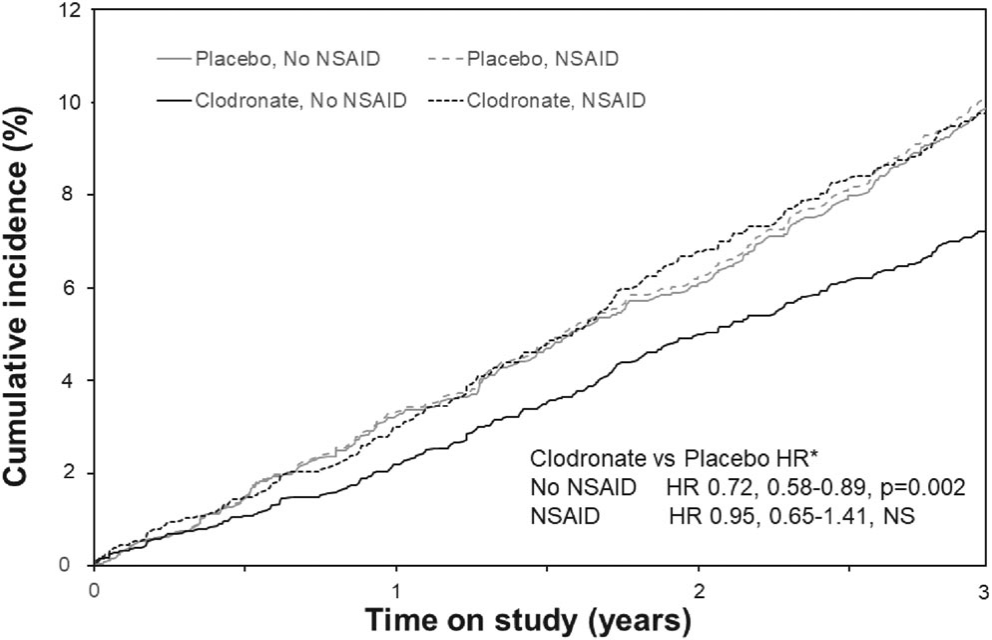
Cumulative incidence of osteoporotic fractures over the 3 years of the study in those women randomized to placebo or clodronate separated into NSAID users and nonusers. HRs derived by Cox regression; HR* are from the adjusted model including age, femoral neck BMD, and weight as covariates. HR = hazard ratio.

A total of 440 women sustained at least one incident osteoporotic fracture during the 3‐year follow‐up period, including 110 women with hip fractures. The mean follow‐up time in NSAID users and nonusers was identical (2.77 years). The numbers of women with incident osteoporotic fractures and hip fractures in NSAID users and nonusers are shown in Table 2, with the cumulative incidence of osteoporotic fractures shown in Fig. 1. Although the crude incidence of osteoporotic fractures was higher in the women using NSAIDs, this was not statistically significant. However, following adjustment for age, femoral neck BMD, weight, osteoarthritis, medication use (asthma/COPD, psycholeptics), sit‐to‐stand difficulty, and treatment group (clodronate/placebo), NSAID use was associated with an increased risk of all osteoporotic fractures, but not in the case of hip fracture alone (hazard ratio [HR] 1.27; 95% confidence interval [CI], 1.01–1.59; HR 0.98; 95% CI, 0.60–1.61, respectively) (Table 2).

**Table 2:** Observed Numbers of Incident Osteoporotic and Hip Fractures in NSAID Users and Nonusers Over the 3 Years of the Study

Fracture outcome	No NSAID		NSAID		NSAID versus no NSAID HR (95% CI), *p*	
	Number	Rate (%)	Number	Rate (%)	Crude	Adjusted
Whole study population						
Osteoporotic	338	8.2	102	9.4	1.17 (0.94–1.46), *p* = 0.172	**1.27 (1.01–1.62), *p* = 0.039**
Hip	90	2.2	20	1.8	0.85 (0.52–1.38), *p* = 0.51	0.96 (0.58–1.59), *p* = 0.88
Placebo group only						
Osteoporotic	198	9.5	51	9.7	1.02 (0.75–1.39), *p* = 0.88	1.11 (0.81–1.52), *p* = 0.52
Hip	49	2.4	8	1.5	0.65 (0.31–1.37), *p* = 0.26	0.73 (0.34–1.57), *p* = 0.42
Clodronate group only						
Osteoporotic	140	6.8	51	9.2	1.37 (1.00–1.89), *p* = 0.053	**1.49 (1.07–2.07), *p* = 0.019**
Hip	41	2.0	12	2.2	1.08 (0.57–2.06), *p* = 0.81	1.23 (0.62–2.43), *p* = 0.55

HRs derived by Cox regression; the adjusted model has included age, femoral neck BMD, weight, osteoarthritis, medication use (asthma/COPD and psycholeptics as separate variables) and sit‐to‐stand test as covariates. The whole study population adjustment also included treatment group (clodronate/placebo) as a covariate. Bold values are significant.

BMD = bone mineral density; CI = confidence interval; COPD = chronic obstructive pulmonary disease; HR = hazard ratio; NSAID = nonsteroidal anti‐inflammatory drug.

Given that treatment with oral clodronate was associated with a significant 23% reduction in osteoporotic fractures, the relationship between NSAID use and fracture was then examined separately in the placebo and clodronate groups (Table 2, Fig. 1). Importantly, although NSAID use was associated with certain clinical characteristics, these and other potential fracture‐related characteristics were well‐balanced between those randomized to receive clodronate or placebo in the presence or absence of NSAID exposure (Table S3). In the placebo group, the rates of osteoporotic and hip fractures did not differ in women receiving NSAIDs compared to those not treated with NSAIDs; eg, the proportion sustaining osteoporotic fractures was 9.5% and 9.7%, respectively (Table 2, Fig. 1) and the HRs for NSAID use were not significant in the crude or adjusted models. In contrast, in the women receiving clodronate, NSAID use was associated with a 37% higher osteoporotic fracture risk, which was of borderline statistical significance; the increase was greater (49%) and statistically significant in the adjusted model (Table 2). Of note, the incidence of osteoporotic fracture in those women on clodronate but also exposed to NSAIDs was similar to that observed in the placebo group (9.2%), with a lower incidence seen in the women not using NSAIDs (6.8%) (Table 2, Fig. 1). The overall reduction in osteoporotic fracture risk seen in the clodronate arm (23%; 95% CI, 7% to 37%; *p* = 0.006), comprised a reduction of 28% (95% CI, 11% to 42%; *p* = 0.002) in women not on NSAIDs, with a nonsignificant 5% reduction (95% CI, −41% to 35%) in those on NSAIDs. The *p* value for the interaction between NSAID use and treatment with clodronate was, however, not significant ranging from 0.18 to 0.20 in the various models.

### NSAID exposure, clodronate, and BMD changes over 3 years

A total of 691 women had repeated measurement of hip and subregional BMD at the end of the 3‐year treatment period. At entry to the study, those that would undergo subsequent repeat measurements were slightly but significantly younger (78.82 ± 3.33 versus 79.61 ± 4.01 years, *p* < 0.001) and had a higher prevalence of mild primary hyperparathyroidism (7.4% versus 1.7%, *p* < 0.001) and vertebral fractures (21.4% versus 13.4%, *p* < 0.001) as these were the inclusion criterion for two observational substudies. There was no significant difference in BMD or weight between those having repeat measurements and those not having repeat BMD; furthermore, the prevalence of NSAID use at baseline and the subsequent exposure to clodronate was similar in the repeat BMD subgroup to that in the whole study population (NSAID use, 22.1% versus 20.5%, *p* = not significant [NS]; and clodronate treatment, 48.6% versus 50.2%, *p* = NS; respectively).

**Fig. 2:**
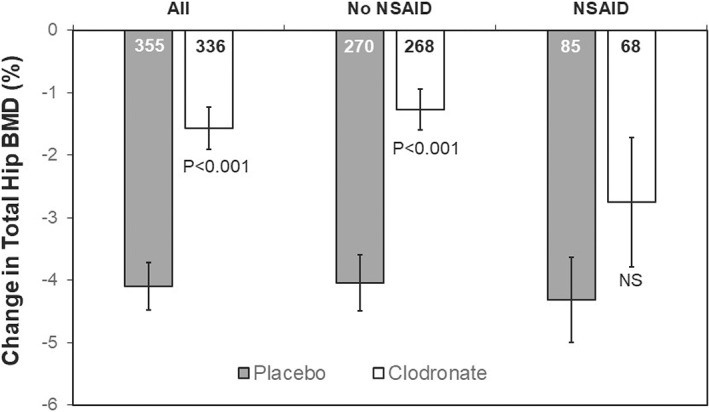
Changes in total hip BMD (mean ± SEM) over 3 years in the subset of women with repeat BMD measurements. The numbers within each bar represent the number of women in each group. Values of *p* are for comparison to BMD changes in the women receiving placebo in the same group for all women, and those receiving or not receiving NSAIDs.

Over the 3 years, the mean decrease in femoral neck and total hip BMD was slightly greater in women reporting NSAID use (*n* = 153) than in nonusers (*n* = 538), but the differences were not statistically significant (femoral neck BMD % change, −3.24 ± 6.93 versus −2.29 ± 6.31, *p* = 0.11; total hip BMD % change, −3.62 ± 7.34 versus −2.66 ± 6.63, *p* = 0.13). When examined in the placebo group alone, the losses in BMD were very similar regardless of exposure to NSAIDs (data for total hip BMD shown in Fig. 2). Overall, treatment with clodronate was associated with a highly significant reduction in the rates of bone loss at the two sites compared to placebo (femoral neck BMD % change, −1.51 ± 6.55 versus −3.44 ± 6.24, *p* < 0.001; total hip BMD % change, −1.57 ± 6.18 versus −4.10 ± 7.14, *p* <0 .001, Fig. 2). However, the prevention of BMD loss by clodronate differed in women with NSAID use compared to those without NSAID use (Fig. 2). The reduction in total hip BMD (and subregions, not shown) in women with NSAID exposure was not significantly different to that observed in the placebo group (Fig. 2); within the women receiving clodronate, the loss of BMD in those receiving NSAIDs was greater than that observed in those not receiving NSAIDs, though this did not quite reach statistical significance (*p* = 0.078) for the total hip. There were no discernible differences in baseline characteristics between women randomized to clodronate in the presence or absence of NSAID use to explain these observations (Table S4).

### NSAID use and compliance with clodronate

To determine whether the observed differences in efficacy of clodronate in the NSAID users and nonusers related to differences in compliance, we compared compliance with clodronate in the two groups. Data on treatment compliance was available in 2529 women (97.0%) on clodronate during the study. Overall, the mean ± SD duration of treatment with clodronate was 2.05 ± 1.10 years, with a median (Q1, Q3) compliance of 74.5% (42.1%, 93.0%) of the provided medication and 44.4% showing high compliance throughout the study period. The mean duration of clodronate treatment was similar in the NSAID users and nonusers (2.04 ± 1.10 years versus 2.05 ± 1.10 years, *p* = NS). The proportion of participants providing clodronate compliance data, and the proportion within them showing high compliance, was similar at each time point over the 3 years of the study in the NSAID users and nonusers (Table S5). Over the entire 3 years of the study, the median (Q1, Q3) clodronate compliance was 71.8% (41.4%, 91.8%) in NSAID users compared to 75.2% (42.5%, 93.3%) in nonusers (*p* = 0.35), and the proportion showing high compliance throughout was 42.4% versus 45.0%, respectively (*p* = 0.31).

## Discussion

This analysis suggests that NSAID use per se has no significant impact on osteoporotic fracture risk. This may reflect the fact that patients prescribed NSAIDs have a number of characteristics usually associated with a reduction in fracture risk, such as younger age, higher weight, and greater BMD. Notably, the use of NSAIDs was also associated with other characteristics that could potentially mediate an increase in falls or fracture risk; eg, although multiple falls in the month prior to study entry was uncommon and balanced between NSAID users and nonusers, there was a significantly higher proportion of patients with rheumatoid arthritis, and NSAID users had lower quadriceps strength and more frequently demonstrated difficulty in the sit‐to‐stand test. The latter two observations may have related to the more frequent reporting of osteoarthritis in NSAID users, an expected finding. Nonetheless, this analysis conducted in a well‐documented clinical study that allows identification and adjustment for multiple confounders suggests that there is little evidence of a direct effect of NSAIDs on fracture risk. Perhaps the most unexpected, but potentially most important finding from a clinical perspective, is the association between NSAID use and apparent complete negation of the protective effect of the oral bisphosphonate, clodronate, on osteoporotic fracture risk. We believe this is the first report of such an interaction; if replicated in studies of other bisphosphonates, this would have a significant impact on the management of individuals at high fracture risk on NSAIDs, and also impact on assumed treatment effect sizes in health economic models for bisphosphonates.

NSAIDs have been widely documented to impact on skeletal metabolism, particularly bone remodeling, in both preclinical and clinical studies.^(^ ^28^, ^29^, ^30^, ^31^, ^32^ ^)^ This has led to many studies of the relationship between NSAID use and osteoporotic fracture risk, but with conflicting results ranging from it being either a protective factor or a risk factor, through to no effect on fracture risk.^(^ ^9^, ^10^, ^11^, ^12^, ^13^, ^14^, ^33^, ^34^ ^)^ The level of documentation in our study and conclusions are similar to those reported from the Study of Osteoporotic Fractures (SOF).^(^ ^14^ ^)^ In the SOF, daily use of aspirin or NSAIDs examined prospectively in a cohort of almost 8000 white women >65 years found that axial BMD was slightly higher in users of either medication compared to nonusers. This relationship persisted even after adjustment for weight, a variety of medications, self‐reported arthritis, and for radiographic findings of osteoarthritis. Despite this, and similar to our results, the risk of all nonspine fractures over 4 years of follow‐up was not different in users compared to nonusers (relative risk 1.0; 95% CI, 0.8–1.2 for both medications).^(^ ^14^ ^)^ In contrast, during prospective follow‐up of the Danish Osteoporosis Prevention Study (DOPS), a partly randomized cohort study of hormone therapy (HT), NSAID use was reported to be associated with a 44% increase in fracture risk, after adjustment for a higher body weight and BMD. In the placebo arm of our study, we likewise saw an increase in fracture risk in the adjusted model, but this was far from statistical significance. The magnitude of effect was much greater in the clodronate treatment arm, suggesting an interaction with treatment efficacy. Whether the relationship of NSAID to fracture risk was independent of HT use was not presented in the DOPS publication.^(^ ^35^ ^)^

Our analysis has several limitations and strengths. Although the test for interaction between treatment and NSAID use was not statistically significant (*p* = 0.18–0.20), the proportion of women taking NSAIDs was somewhat smaller than those not exposed to NSAIDs, so that the analysis is underpowered to detect a true difference, a factor that should be borne in mind in interpretation of the interaction analysis. This was a post hoc analysis of a study not designed to address this particular question; nonetheless, the high quality and wide‐ranging data capture permitted a much more nuanced analysis of the effects of NSAIDs on fracture risk. Our study population comprised elderly, community‐dwelling women, and the findings might not be translatable into men or younger individuals of either sex, though biological reasons for potential differences are hard to identify. Our analysis was based on self‐reported NSAID use at entry to the study, though this was verified against GP prescriptions where available. The clinical, biochemical, and hematological characteristics of the NSAID users at entry were in keeping with those that might be expected in those exposed to such medications (eg, more osteoarthritis, lower hemoglobin, increased platelets, higher serum creatinine). It is, of course, possible that exposures to NSAIDs might have altered during the trial, but NSAID use was documented to be present for over several years in the users in this study, suggesting relative stability of exposure. It is unlikely that many of those not taking NSAIDs were subsequently prescribed them given that the average age of the women was 79 years at entry and guidance recommends relative avoidance of such drugs at older ages. Finally, although the observed effect of NSAIDs reducing the effect of the oral bisphosphonate clodronate on fracture risk could be a chance observation, the fact that an effect was also observed on a second outcome measure, BMD, suggests that the phenomenon is worthy of exploration in other clinical trials.

The mechanism(s) by which NSAID use might reduce the efficacy of clodronate can only be speculative at this point. The lack of convincing evidence for a significant effect of NSAIDs themselves on bone loss or fracture risk in our and other cohorts makes consideration of other potential mechanisms necessary, particularly those arising outside the skeleton. For example, NSAID use might be associated with decreased adherence to the bisphosphonate or might impair its intestinal absorption. Within this RCT, we have been able to examine the impact of NSAIDs on compliance with clodronate, and while the median intake and proportion showing high compliance were slightly lower in NSAID users, this did not reach statistical significance and is highly unlikely to explain the observations. Oral clodronate has generally higher bioavailability (1% to 2% intestinal absorption)^(^ ^36^ ^)^ compared to that of oral nitrogen‐containing bisphosphonates such as alendronate (<1%),^(^ ^37^ ^)^ but the absorption is still very low. Advice to take oral bisphosphonates on an empty stomach, washed down with water only at least 30 minutes before food and other medications recognizes that this absorption can easily be reduced further. This is especially emphasized for the use of other medications such as calcium supplements and antacids, some of which contain calcium and similar ions, whereas others alter acidity through other mechanisms. Despite the expectation that agents that reduce the secretion of gastric acid and elevate gastric pH might increase bisphosphonate bioavailability,^(^ ^37^ ^)^ studies that have compared fracture rates in bisphosphonate users with or without concomitant exposure to PPI medications have shown inconsistent results.^(^ ^38^, ^39^, ^40^ ^)^ In a post hoc analysis of risedronate randomized, controlled studies, concomitant treatment with PPIs showed no interaction with the bisphosphonates efficacy to reduce vertebral fractures.^(^ ^41^ ^)^ In contrast, in a retrospective, observational study from the General Practice Research Database, fracture rates were higher in those patients using PPIs and bisphosphonates compared to bisphosphonates alone, though adjustment for potential confounding was limited.^(^ ^38^ ^)^ Nonetheless, although the type of antacid used differed slightly between NSAID users and nonusers in our study, overall antacid exposure was similar in the two groups and adjustment for this had no significant impact on the association between NSAID use and impaired efficacy of clodronate.

In summary, although we found little evidence for NSAID use as a risk factor for incident osteoporotic fractures among elderly community‐dwelling women, the observation that NSAID use significantly reduced the ability of clodronate to prevent bone loss and fractures is unique and of high clinical importance. The marked reduction in efficacy does not appear to be mediated by imbalances in baseline characteristics or lower compliance. Although possible mechanisms need further exploration, there is an urgent need to examine this interaction in studies of other bisphosphonates in osteoporosis and other bone diseases.

## AUTHOR CONTRIBUTIONS

**Zhangan Zheng:** Conceptualization; data curation; formal analysis; writing – original draft; writing – review and editing. **Helena Johansson:** Formal analysis; writing – original draft; writing – review and editing. **Nicholas C. Harvey:** Writing – review and editing. **Mattias Lorentzon:** Writing – review and editing. **Liesbeth Vandenput:** Writing – review and editing. **Enwu Liu** **:** Writing – review and editing. **John A. Kanis:** Writing – review and editing. **Eugene McCloskey:** Conceptualization; formal analysis; supervision; writing – review and editing.

### Peer Review

The peer review history for this article is available at https://publons.com/publon/10.1002/jbmr.4548.

## Supporting information


Table S1
Table S2
Table S3
Table S4
Table S5

## Data Availability

The data that support the findings of this study are available from the corresponding author upon reasonable request.
