# Supplementary material for: Potential Adverse Effect of Nonsteroidal Anti‐Inflammatory Drugs (NSAIDs) on Bisphosphonate Efficacy: An Exploratory Post Hoc Analysis From a Randomized Controlled Trial of Clodronate
Source: J Bone Miner Res. 2022 Apr 20;37(6):1117–24. doi: 10.1002/jbmr.4548 (PMC9487988; doi:10.1002/jbmr.4548)
Supplement: Supplementary file 1 — Table S1 Table S2 Table S3 Table S4 Table S5 [file JBMR-37-1117-s001.docx]

**Supplementary material**

#### Table S1. Baseline characteristics of the study population (5212 elder women) with and without NSAIDs use

| Variable | No NSAID (n=4130) | NSAID (n=1082) | P value |
| --- | --- | --- | --- |
| **Cardiovascular parameters (mean ± SD)** | | | |
| Lying SBP (mmHg) | 150.24 ± 22.72 | 152.90 ± 23.15 | 0.001 |
| Lying DBP (mmHg) | 79.40 ± 12.13 | 80.73 ± 12.16 | 0.001 |
| Lying Pulse (bpm) | 75.84 ± 9.76 | 74.94± 9.79 | 0.007 |
| Standing SBP (mmHg) | 143.16 ± 22.93 | 145.74 ± 23.11 | 0.001 |
| Standing DBP (mmHg) | 75.79 ± 12.35 | 76.94 ± 12.22 | 0.008 |
| Standing Pulse (bpm) | 79.65 ± 9.89 | 79.19 ± 9.52 | 0.184 |
| **Medical history N(%)** |  |  |  |
| Stroke | 102 (2.5) | 25 (2.3) | 0.825 |
| Insulin-dependent diabetes | 30 (0.7) | 10 (0.9) | 0.556 |
| Insulin-independent diabetes | 188 (4.6) | 43 (4.0) | 0.455 |
| Parkinson’s Disease | 36 (0.9) | 7 (0.6) | 0.467 |
| Osteoarthritis | 2755 (66.7) | 892 (82.4) | 0.000 |
| Rheumatoid arthritis | 86 (2.1) | 48 (4.4) | 0.000 |
| [Hypothyroidism](https://www.google.com.hk/search?safe=strict&hl=zh-CN&biw=1399&bih=764&q=Hypothyroidism&sa=X&ved=2ahUKEwjBgcj1sZvnAhWCyosBHYvGAacQ7xYoAHoECAwQJg) | 341 (8.3) | 92 (8.5) | 0.805 |
| Hyperthyroidism | 33 (0.8) | 16 (1.5) | 0.050 |
| **Medication use N (%)** |  |  |  |
| Calcium | 607 (14.7) | 185 (17.1) | 0.05 |
| Vitamin D | 1879 (45.5) | 548 (50.6) | 0.002 |
| Estrogen | 83 (2.0) | 21 (1.9) | 0.885 |
| Didronel PMO | 16 (0.4) | 2 (0.2) | 0.312 |
| Thyroxine | 361 (8.7) | 105 (9.7) | 0.323 |
| Corticoids | 402 (9.7) | 98 (9.1) | 0.501 |
| Anabolic | 7 (0.2) | 1 (0.1) | 0.564 |

**SBP – systolic blood pressure; DBP diastolic blood pressure**

#### Table S2. Baseline blood routine and biochemical parameters of the population stratified by NSAIDs use (mean ± SD)

| Variable | No NSAID (n=4130) | NSAID (n=1082) | p value |
| --- | --- | --- | --- |
| Age (years) | 79.58 ± 4.02 | 79.19 ± 3.62 | 0.004 |
| Calcium (mmol/L) | 2.41 ± 0.12 | 2.40 ± 0.11 | 0.275 |
| Phosphate (mmol/L) | 1.07 ± 0.14 | 1.07 ± 0.14 | 0.660 |
| Creatinine (μmol/L) | 105 ± 18 | 107 ± 21 | 0.000 |
| AST (IU/L) | 20.83 ± 5.95 | 20.89 ± 6.20 | 0.774 |
| ALT (IU/L) | 16.35 ± 7.77 | 17.11 ± 8.70 | 0.005 |
| Albumin (g/dL) | 42.27±2.21 | 42.24±2.22 | 0.670 |
| Bilirubin (mg/dl) | 9.49 ± 4.25 | 8.78 ± 4.57 | 0.000 |
| GGT (IU/L) | 29.60 ± 47.68 | 29.27 ± 41.15 | 0.835 |
| Haemoglobin (g/dL) | 13.48 ± 1.14 | 13.19 ± 1.24 | 0.000 |
| White cell count (× 10*^9^*/L) | 6.73 ± 2.31 | 6.94 ± 2.33 | 0.006 |
| Platelets (× 10*^9^*/L) | 242.17± 60.79 | 249.37 ± 63.16 | 0.001 |
| Red cell count (× 10*^12^* /L) | 4.41±0.38 | 4.32±0.39 | 0.000 |
| Mean Corpuscular Volume (fl) | 91.06 ± 5.00 | 90.87 ± 5.44 | 0.273 |
| Haematocrit (%) | 0.40 ± 0.03 | 0.39 ± 0.04 | 0.000 |
| MCH (pg) | 30.65 ± 1.92 | 30.56 ± 2.13 | 0.188 |
| MCHC (g/dL) | 33.62 ± 0.67 | 33.59 ± 0.71 | 0.173 |
| Neutrophils (× 10*^9^*/L) | 4.08 ± 1.43 | 4.28 ± 1.58 | 0.000 |
| Lymphocytes (× 10*^9^*/L) | 1.87 ± 1.49 | 1.86 ± 1.38 | 0.809 |
| Monocytes (× 10*^9^*/L) | 0.55 ± 0.28 | 0.55 ± 0.20 | 0.997 |
| Eosinophils (× 10*^9^*/L) | 0.17 ± 0.14 | 0.20 ± 0.15 | 0.000 |
| Basophils (× 10*^9^*/L) | 0.06 ± 0.09 | 0.06 ± 0.04 | 0.681 |
| ESR (mm/h) | 16.32 ± 11.97 | 16.59 ± 11.49 | 0.508 |

AST aspartate transaminase; ALT alanine transaminase; GGT gamma-glutamyl transferase; MCH mean corpuscular haemoglobin; MCHC mean corpuscular haemoglobin concentration; ESR erythrocyte sedimentation rate

#### Table S3. Selected baseline characteristics of the participants randomised to clodronate or placebo in the NSAID nonusers and users stratified by NSAIDs use (mean ± SD unless specified). Within the users and non-users there were no significant differences in baseline characteristics between those randomised to placebo or clodronate.

|  | **No NSAID** | | **NSAID** | |
| --- | --- | --- | --- | --- |
| **Baseline characteristic** | **Placebo** | **Clodronate** | **Placebo** | **Clodronate** |
| Age (years) | 79.59 ± 3.96 | 79.57 ± 4.07 | 79.31 ± 3.79 | 79.08 ± 3.45 |
| Height (m) | 1.55 ± 0.06 | 1.55 ± 0.06 | 1.56 ± 0.06 | 1.55 ± 0.06 |
| Weight (kg) | 64.31 ± 11.92 | 65.02 ± 12.18 | 66.70 ± 12.20 | 66.72 ± 12.05 |
| BMI (kg/m²) | 26.42 ± 4.53 | 26.74 ± 4.76 | 27.41 ± 4.67 | 27.58 ± 4.79 |
| **Biochemistry** | | | | |
| Total ALP (IU/l) | 208 ± 83 | 205 ± 72 | 210 ± 77 | 209 ± 63 |
| PINP (ng/ml) | 62.3 ± 33.3 | 62.7 ± 33.5 | 69.2 ± 43.3 | 64.9 ± 35.0 |
| CTX (ng/ml) | 0.40 ± 0.20 | 0.40 ± 0.21 | 0.42 ± 0.22 | 0.39 ± 0.21 |
| Creatinine (μmol/L) | 104 ± 18 | 105 ± 18 | 107 ± 21 | 107 ± 20 |
| eGFR<30ml/min/1.73m^2^ (n,%) | 65 (3.1) | 76 (3.7) | 29 (5.5) | 30 (5.4) |
| **Medications of interest** | | | | |
| Any antacid use (n,%) | 539 (25.9) | 519 (25.3) | 136 (25.8) | 145 (26.1) |
| PPI use (n,%) | 184 (8.9) | 179 (8.7) | 40 (7.6) | 33 (5.9) |
| Psycholeptic use (n,%) | 487 (23.4) | 449 (21.9) | 153 (29.0) | 138 (24.9) |
| **Other conditions/risk factors** | | | | |
| Primary hyperparathyroidism (n,%) | 54 (2.6) | 57 (2.8) | 4 (0.8) | 11 (2.0) |
| Osteoarthritis (n,%) | 1381 (66.7) | 1368 (66.7) | 442 (83.9) | 450 (81.1) |
| Prior fracture (n,%) | 930 (45.3) | 924 (45.4) | 233 (44.4) | 270 (49.1) |
| Glucocorticoid (n,%) | 193 (9.3) | 209 (10.2) | 46 (8.7) | 52 (9.4) |
| **DXA and Muscle strength** | | | | |
| FN-BMD (g/cm^2^) | 0.64 ± 0.12 | 0.64 ± 0.12 | 0.66 ± 0.12 | 0.66 ± 0.12 |
| Quadriceps strength (N) | 127.0 ± 60.4 | 127.8 ± 60.0 | 121.3 ± 54.1 | 120.9 ± 56.5 |

#### Table S4. Selected baseline characteristics of the participants in the repeat BMD sub-group randomised to clodronate in the absence or presence of NSAID use (mean ± SD unless specified). There were no statistically significant differences between the groups.

| **Characteristic** | **No NSAID (n=268)** | **NSAID (n=68)** |
| --- | --- | --- |
| Age (y) | 78.8 ± 3.4 | 78.3 ± 2.8 |
| BMI (kg/m^2^) | 27.1 ± 4.5 | 27.2 ± 4.7 |
| **Biochemistry** | | |
| Creatinine (μmol/l) | 100 ± 17 | 101 ± 18 |
| eGFR<30ml/min/1.73m^2^ | 4 (1.5) | 1 (1.5) |
| Total ALP (IU/l) | 198 ± 66 | 197 ± 52 |
| PINP (ng/ml) | 60.4 ± 29.3 | 59.7 ± 25.0 |
| CTX (ng/ml) | 0.43 ± 0.22 | 0.40 ± 0.21 |
| **Medications of interest** | | |
| Any antacid use (n,%) | 59 (22.0) | 16 (23.5) |
| PPI use (n,%) | 23 (8.6) | 4 (5.9) |
| **Other conditions/risk factors** | | |
| Hyperparathyroidism (n,%) | 27 (10.1) | 3 (4.4) |
| Prior fractures (n,%) | 110 (41.8) | 37 (54.4) |
| Glucocorticoid use (n,%) | 21 (7.8) | 4 (5.9) |
| FN-BMD (g/cm^2^) | 0.64 ± 0.11 | 0.66 ± 0.13 |

#### Table S5. Compliance within each 6-month study period for the participants receiving clodronate separated into NSAID nonusers and users. Compliance with clodronate was similar in both NSAID users and non-users.

|  | No NSAID (n=2051) | | NSAID (n=555) | |
| --- | --- | --- | --- | --- |
| Month | Proportion reporting^a^ (%) | High Compliance^b^ (%) | Proportion reporting^a^ (%) | High Compliance^b^ (%) |
| 6 | 92.8 | 61.4 | 95.3 | 58.6 |
| 12 | 70.5 | 64.9 | 72.8 | 62.4 |
| 18 | 63.0 | 65.2 | 61.4 | 66.6 |
| 24 | 57.8 | 67.3 | 57.7 | 67.2 |
| 30 | 54.6 | 68.5 | 54.2 | 68.4 |
| 36 | 50.5 | 67.0 | 50.8 | 68.4 |

^a^The proportion of the initial number randomised to the group reporting compliance data at the time point shown (e.g month 12 represents those reporting compliance at that time point for the 6-12 month period).

^b^The proportion of those reporting at any time point who had returned less than 20% of allocated medication (i.e. deemed to have high compliance of >80% of allocated medication).
